# 2-(4-Chloro­phen­yl)naphtho­[1,8-*de*][1,3,2]diaza­borinane

**DOI:** 10.1107/S1600536811025487

**Published:** 2011-07-02

**Authors:** Matthew P. Akerman, Ross S. Robinson, Cathryn A. Slabber

**Affiliations:** a University of KwaZulu-Natal, School of Chemistry, Private Bag XO1, Scottsville, Pietermaritzburg 3209, South Africa

## Abstract

The title compound, C_16_H_12_BClN_2_, is one in a series of diaza­borinanes, derived from 1,8-diaminona­phthalene, featuring substitution at the 1, 2 and 3 positions in the nitro­gen-boron heterocycle. The structure deviates from planarity, the torsion angle subtended by the *p*-chloro­phenyl ring relative to the nitro­gen–boron heterocycle being −44-.3(3)°. The mol­ecules form infinite chains with strong inter­actions between the vacant *pz* orbital of the B atom and the π-system of an adjacent mol­ecule. The distance between the B atom and the 10-atom centroid of an adjacent naphthalene ring is 3.381 (4) Å. One N-H H atom is weakly hydrogen bonded to the Cl atom of an adjacent mol­ecule. This combination of inter­molecular inter­actions leads to the formation of an infinite two-dimensional network perpendic­ular to the *c* axis.

## Related literature

For the synthesis of related compounds, see: Letsinger & Hamilton (1958 ▶); Pailer & Fenzl (1961 ▶); Kaupp *et al.* (2003 ▶); Slabber 2011 ▶. For single-crystal X-ray structures and lumin­escence studies of related compounds, see: Weber, *et al.* (2009 ▶).
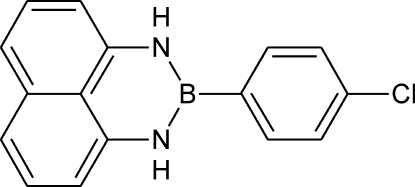


## Experimental

### 

#### Crystal data

C_16_H_12_BClN_2_
*M* *_r_* = 278.54
Monoclinic, 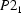

*a* = 4.7165 (2) Å
*b* = 10.2815 (4) Å
*c* = 13.5711 (6) Å
β = 96.555 (4)°
*V* = 653.79 (5) Å^3^
*Z* = 2
Mo *K*α radiation
μ = 0.28 mm^−1^
*T* = 296 K
0.50 × 0.15 × 0.07 mm

#### Data collection

Oxford Diffraction Xcalibur 2 CCD diffractometer
Absorption correction: multi-scan (*SADABS*; Sheldrick, 2003 ▶) *T*_min_ = 0.896, *T*_max_ = 0.981
4902 measured reflections
2286 independent reflections
2011 reflections with *I* > 2σ(*I*)
*R*_int_ = 0.027

#### Refinement

*R*[*F* ^2^ > 2σ(*F* ^2^)] = 0.039
*wR*(*F* ^2^) = 0.098
*S* = 1.00
2286 reflections
189 parameters
1 restraint
H atoms treated by a mixture of independent and constrained refinement
Δρ_max_ = 0.15 e Å^−3^
Δρ_min_ = −0.25 e Å^−3^
Absolute structure: Flack (1983 ▶), 924 Friedel pairs
Flack parameter: 0.05 (7)

### 

Data collection: *CrysAlis CCD* (Oxford Diffraction, 2008 ▶); cell refinement: *CrysAlis CCD*; data reduction: *CrysAlis RED* (Oxford Diffraction, 2008 ▶); program(s) used to solve structure: *SHELXS97* (Sheldrick, 2008 ▶); program(s) used to refine structure: *SHELXL97* (Sheldrick, 2008 ▶); molecular graphics: *WinGX* (Farrugia, 1999 ▶); software used to prepare material for publication: *WinGX* (Farrugia, 1999 ▶).

## Supplementary Material

Crystal structure: contains datablock(s) I, global. DOI: 10.1107/S1600536811025487/om2441sup1.cif

Structure factors: contains datablock(s) I. DOI: 10.1107/S1600536811025487/om2441Isup2.hkl

Supplementary material file. DOI: 10.1107/S1600536811025487/om2441Isup3.cml

Additional supplementary materials:  crystallographic information; 3D view; checkCIF report

## Figures and Tables

**Table 1:** Hydrogen-bond geometry (Å, °)

*D*—H⋯*A*	*D*—H	H⋯*A*	*D*⋯*A*	*D*—H⋯*A*
N2—H2⋯Cl^i^	0.78 (2)	2.93 (2)	3.666 (2)	158 (2)

Symmetry code: (i) 
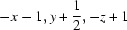
.

**Fig. 1.**
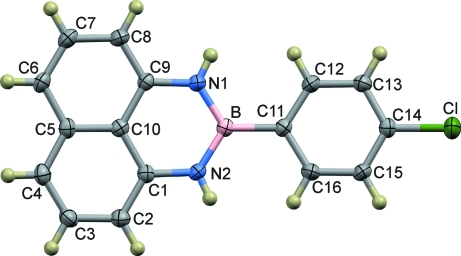
Thermal ellipsoid plot of 2-(4-chlorophenyl)-naphtho[1,8-de][1,3,2]diazaborinane (50% probability surfaces). Hydrogen atoms have been rendered as spheres of arbitrary radius.

**Fig. 2.**
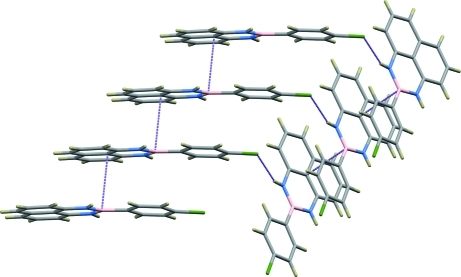
Two-dimensional network of the title compound perpendicular to the c axis. The network is supported by B···π interactions and hydrogen bonds between the terminal chlorine atom and the N-H hydrogen, as indicated.

## supplementary crystallographic information

### Comment

The structure of the title compound is nominally planar with a slight rotation of the *p*-chlorophenyl ring relative to the naphthalene rings and boron-nitrogen heterocycle. The N1—B—C11—C12 torsion angle is -4.3 (3)° (refer to Figure 1 for the atom numbering scheme). The orientation of the heterocycle relative to the diazaborolyl groups is critical, since as the rings approach co-planarity there is more effective overlap of the π-systems of the boron atom and the carbon atom to which it is attached. The bond lengths N1—B and N2—B are approximately equal, measuring 1.416 (3) and 1.405 (3) Å, respectively, while the B—C11 bond length is 1.568 (4) Å. The Cl—C14 bond length is 1.736 (2) Å. The N1—B—N2 bond angle is 115.6 (2)°, the N1—B—C11 and N2—B—C11 bond angles are equal, both measuring 122.2 (2)°. These bond length and angles are comparable to those of previously reported diazaborolyl systems (Weber *et al.*, 2009).

Examination of the title compound showed that there is a short contact between the boron atom and the naphthalene rings of an adjacent molecule. The distance from the boron atom to the 10-atom naphthalene centroid is 3.381 (4) Å. These B-π interaction link the molecules, forming infinite, one-dimensional chains. Adjacent one dimensional chains are then weakly hydrogen-bonded together by a N-H hydrogen atom and the chlorine atom of the adjacent molecule. These hydrogen bonds are likely to be very weak as they are only nominally shorter than the sum of the van der Waals radii (0.022 Å shorter) (Table 1). The combination of intermolecular interactions results in the formation of infinite, two-dimensional sheets (Figure 2). The two-dimensional sheet runs perpendicular to the *c* axis.

### Experimental

To a solution of 1,8-diaminonaphthalene in toluene (4.11 mmol in 50 ml, 0.82*M*) (Letsinger & Hamilton, 1958; Slabber, 2011) was added the 3-chlorophenylboronic acid (4.11 mmol) in one portion. The round-bottomed flask was equipped with a Dean and Stark trap, and the solution was stirred and heated to reflux for 3 h. The solvent was removed *in vacuo* and column chromatography of the crude solid using silica gel as the stationary phase and eluting with CH_2_Cl_2_ yielded pale green crystalline material upon evaporation of the eluent with a yield of 66%. Recrystallization of the material from dichloromethane yielded crystals suitable for single-crystal X-ray diffraction analysis were grown.

### Refinement

The positions of all hydrogen atoms were calculated using the standard riding model of *SHELXL97.* with C—H(aromatic) distances of 0.93 Å and *U*_iso_ = 1.2 *U*_eq_. The only exception is the NH hydrogen atoms which were located in the difference Fourier map and allowed to refine isotropically.

### Crystal data

**Table:**

C_16_H_12_BClN_2_	*F*(000) = 288
*M**_r_* = 278.54	*D*_x_ = 1.415 Mg m^−^^3^
Monoclinic, *P*2_1_	Mo *K*α radiation, λ = 0.71073 Å
Hall symbol: P 2yb	Cell parameters from 2982 reflections
*a* = 4.7165 (2) Å	θ = 3.6–32.0°
*b* = 10.2815 (4) Å	µ = 0.28 mm^−^^1^
*c* = 13.5711 (6) Å	*T* = 296 K
β = 96.555 (4)°	Plate, colourless
*V* = 653.79 (5) Å^3^	0.50 × 0.15 × 0.07 mm
*Z* = 2	

### Data collection

**Table:**

Oxford Diffraction Xcalibur 2 CCD diffractometer	2286 independent reflections
Radiation source: fine-focus sealed tube	2011 reflections with *I* > 2σ(*I*)
graphite	*R*_int_ = 0.027
Detector resolution: 8.4190 pixels mm^-1^	θ_max_ = 26.1°, θ_min_ = 3.6°
ω scans	*h* = −5→5
Absorption correction: multi-scan (*SADABS*; Sheldrick, 2003)	*k* = −12→11
*T*_min_ = 0.896, *T*_max_ = 0.981	*l* = −16→16
4902 measured reflections	

### Refinement

**Table:**

Refinement on *F*^2^	Secondary atom site location: difference Fourier map
Least-squares matrix: full	Hydrogen site location: inferred from neighbouring sites
*R*[*F*^2^ > 2σ(*F*^2^)] = 0.039	H atoms treated by a mixture of independent and constrained refinement
*wR*(*F*^2^) = 0.098	*w* = 1/[σ^2^(*F*_o_^2^) + (0.0669*P*)^2^] where *P* = (*F*_o_^2^ + 2*F*_c_^2^)/3
*S* = 1.00	(Δ/σ)_max_ < 0.001
2286 reflections	Δρ_max_ = 0.15 e Å^−^^3^
189 parameters	Δρ_min_ = −0.25 e Å^−^^3^
1 restraint	Absolute structure: Flack (1983), 924 Friedel pairs
Primary atom site location: structure-invariant direct methods	Flack parameter: 0.05 (7)

### Special details

**Table:**

Geometry. All esds (except the esd in the dihedral angle between two l.s. planes) are estimated using the full covariance matrix. The cell esds are taken into account individually in the estimation of esds in distances, angles and torsion angles; correlations between esds in cell parameters are only used when they are defined by crystal symmetry. An approximate (isotropic) treatment of cell esds is used for estimating esds involving l.s. planes.
Refinement. Refinement of *F*^2^ against ALL reflections. The weighted *R*-factor *wR* and goodness of fit *S* are based on *F*^2^, conventional *R*-factors *R* are based on *F*, with *F* set to zero for negative *F*^2^. The threshold expression of *F*^2^ > 2σ(*F*^2^) is used only for calculating *R*-factors(gt) *etc*. and is not relevant to the choice of reflections for refinement. *R*-factors based on *F*^2^ are statistically about twice as large as those based on *F*, and *R*- factors based on ALL data will be even larger.

### Fractional atomic coordinates and isotropic or equivalent isotropic displacement parameters (Å^2^)

**Table:**

	*x*	*y*	*z*	*U*_iso_*/*U*_eq_	
Cl	−1.03675 (12)	0.41269 (9)	0.42497 (4)	0.0546 (2)	
N1	−0.1813 (4)	0.7869 (2)	0.15026 (15)	0.0391 (4)	
H1	−0.257 (5)	0.732 (3)	0.1114 (18)	0.048 (8)*	
N2	−0.1196 (4)	0.9013 (2)	0.30427 (13)	0.0398 (4)	
H2	−0.134 (5)	0.911 (3)	0.3607 (17)	0.047 (7)*	
C1	0.0738 (5)	0.9863 (2)	0.26954 (16)	0.0362 (5)	
C2	0.2031 (5)	1.0847 (2)	0.32771 (18)	0.0462 (6)	
H2A	0.1624	1.0952	0.3927	0.055*	
C3	0.3950 (6)	1.1682 (2)	0.28863 (19)	0.0498 (6)	
H3	0.4782	1.2354	0.3278	0.060*	
C4	0.4629 (5)	1.1536 (2)	0.19447 (19)	0.0479 (6)	
H4	0.5938	1.2097	0.1706	0.057*	
C5	0.3366 (5)	1.0540 (2)	0.13264 (16)	0.0387 (5)	
C6	0.4036 (5)	1.0340 (2)	0.03481 (17)	0.0447 (5)	
H6	0.5346	1.0879	0.0087	0.054*	
C7	0.2761 (5)	0.9356 (3)	−0.02132 (16)	0.0489 (6)	
H7	0.3219	0.9238	−0.0856	0.059*	
C8	0.0804 (5)	0.8527 (2)	0.01465 (17)	0.0463 (6)	
H8	−0.0039	0.7867	−0.0254	0.056*	
C9	0.0105 (4)	0.8681 (2)	0.11029 (16)	0.0359 (5)	
C10	0.1391 (4)	0.9690 (2)	0.17085 (16)	0.0350 (5)	
C11	−0.4634 (5)	0.7024 (2)	0.29241 (16)	0.0358 (5)	
C12	−0.6037 (5)	0.6040 (2)	0.23650 (17)	0.0440 (6)	
H11	−0.5771	0.5972	0.1698	0.053*	
C13	−0.7810 (5)	0.5156 (2)	0.27541 (17)	0.0455 (6)	
H12	−0.8725	0.4510	0.2357	0.055*	
C14	−0.8199 (4)	0.5247 (2)	0.37392 (17)	0.0389 (5)	
C15	−0.6900 (5)	0.6218 (3)	0.43220 (17)	0.0471 (6)	
H14	−0.7205	0.6287	0.4985	0.057*	
C16	−0.5137 (5)	0.7092 (3)	0.39146 (17)	0.0485 (6)	
H15	−0.4260	0.7746	0.4314	0.058*	
B	−0.2545 (5)	0.7993 (3)	0.24819 (18)	0.0346 (5)	

### Atomic displacement parameters (Å^2^)

**Table:**

	*U*^11^	*U*^22^	*U*^33^	*U*^12^	*U*^13^	*U*^23^
Cl	0.0573 (3)	0.0516 (3)	0.0563 (3)	−0.0125 (3)	0.0131 (2)	0.0070 (3)
N1	0.0428 (10)	0.0405 (11)	0.0344 (10)	−0.0057 (9)	0.0062 (8)	−0.0030 (9)
N2	0.0443 (9)	0.0446 (11)	0.0319 (9)	−0.0039 (9)	0.0107 (7)	−0.0019 (11)
C1	0.0350 (10)	0.0339 (12)	0.0398 (11)	0.0027 (9)	0.0046 (9)	0.0023 (9)
C2	0.0519 (14)	0.0451 (14)	0.0420 (13)	−0.0015 (11)	0.0071 (11)	−0.0053 (11)
C3	0.0554 (15)	0.0415 (15)	0.0511 (16)	−0.0086 (11)	0.0005 (12)	−0.0048 (11)
C4	0.0506 (14)	0.0385 (14)	0.0547 (15)	−0.0025 (11)	0.0065 (11)	0.0089 (11)
C5	0.0360 (11)	0.0359 (12)	0.0437 (12)	0.0038 (9)	0.0019 (9)	0.0091 (10)
C6	0.0490 (13)	0.0469 (13)	0.0395 (13)	0.0008 (11)	0.0109 (10)	0.0118 (11)
C7	0.0551 (13)	0.0576 (18)	0.0356 (11)	0.0042 (11)	0.0123 (10)	0.0064 (11)
C8	0.0534 (14)	0.0521 (14)	0.0342 (12)	−0.0029 (11)	0.0087 (10)	−0.0059 (11)
C9	0.0336 (10)	0.0392 (12)	0.0353 (11)	0.0029 (8)	0.0053 (8)	0.0033 (9)
C10	0.0343 (10)	0.0342 (11)	0.0361 (11)	0.0072 (9)	0.0020 (9)	0.0033 (9)
C11	0.0354 (11)	0.0367 (12)	0.0354 (11)	0.0040 (9)	0.0051 (8)	0.0037 (9)
C12	0.0521 (14)	0.0473 (15)	0.0337 (12)	−0.0039 (11)	0.0094 (11)	−0.0010 (10)
C13	0.0524 (13)	0.0437 (14)	0.0397 (13)	−0.0071 (11)	0.0023 (10)	−0.0035 (10)
C14	0.0354 (11)	0.0378 (12)	0.0435 (13)	0.0018 (9)	0.0047 (9)	0.0058 (10)
C15	0.0564 (14)	0.0538 (15)	0.0325 (12)	−0.0100 (12)	0.0108 (10)	−0.0030 (11)
C16	0.0550 (15)	0.0512 (15)	0.0399 (13)	−0.0140 (12)	0.0074 (11)	−0.0092 (12)
B	0.0319 (11)	0.0361 (13)	0.0358 (12)	0.0030 (10)	0.0037 (9)	0.0049 (10)

### Geometric parameters (Å, °)

**Table:**

Cl—C14	1.736 (2)	C6—H6	0.9300
N1—C9	1.386 (3)	C7—C8	1.386 (3)
N1—B	1.416 (3)	C7—H7	0.9300
N1—H1	0.82 (3)	C8—C9	1.384 (3)
N2—C1	1.384 (3)	C8—H8	0.9300
N2—B	1.405 (3)	C9—C10	1.416 (3)
N2—H2	0.78 (2)	C11—C12	1.387 (3)
C1—C2	1.382 (3)	C11—C16	1.393 (3)
C1—C10	1.419 (3)	C11—B	1.568 (3)
C2—C3	1.396 (3)	C12—C13	1.380 (3)
C2—H2A	0.9300	C12—H11	0.9300
C3—C4	1.361 (3)	C13—C14	1.373 (3)
C3—H3	0.9300	C13—H12	0.9300
C4—C5	1.411 (3)	C14—C15	1.373 (3)
C4—H4	0.9300	C15—C16	1.382 (3)
C5—C6	1.414 (3)	C15—H14	0.9300
C5—C10	1.418 (3)	C16—H15	0.9300
C6—C7	1.364 (3)		
			
C9—N1—B	123.6 (2)	C7—C8—H8	120.0
C9—N1—H1	114.6 (17)	C8—C9—N1	122.3 (2)
B—N1—H1	121.7 (17)	C8—C9—C10	119.74 (19)
C1—N2—B	124.20 (18)	N1—C9—C10	117.99 (18)
C1—N2—H2	113 (2)	C9—C10—C5	119.67 (19)
B—N2—H2	123 (2)	C9—C10—C1	120.99 (19)
C2—C1—N2	122.2 (2)	C5—C10—C1	119.3 (2)
C2—C1—C10	120.1 (2)	C12—C11—C16	116.2 (2)
N2—C1—C10	117.69 (19)	C12—C11—B	122.39 (18)
C1—C2—C3	119.8 (2)	C16—C11—B	121.4 (2)
C1—C2—H2A	120.1	C13—C12—C11	122.9 (2)
C3—C2—H2A	120.1	C13—C12—H11	118.5
C4—C3—C2	121.4 (2)	C11—C12—H11	118.5
C4—C3—H3	119.3	C14—C13—C12	118.8 (2)
C2—C3—H3	119.3	C14—C13—H12	120.6
C3—C4—C5	120.7 (2)	C12—C13—H12	120.6
C3—C4—H4	119.7	C13—C14—C15	120.7 (2)
C5—C4—H4	119.7	C13—C14—Cl	119.54 (18)
C4—C5—C6	122.6 (2)	C15—C14—Cl	119.78 (18)
C4—C5—C10	118.7 (2)	C14—C15—C16	119.4 (2)
C6—C5—C10	118.7 (2)	C14—C15—H14	120.3
C7—C6—C5	120.1 (2)	C16—C15—H14	120.3
C7—C6—H6	120.0	C15—C16—C11	122.0 (2)
C5—C6—H6	120.0	C15—C16—H15	119.0
C6—C7—C8	121.9 (2)	C11—C16—H15	119.0
C6—C7—H7	119.1	N2—B—N1	115.57 (19)
C8—C7—H7	119.1	N2—B—C11	122.17 (18)
C9—C8—C7	120.0 (2)	N1—B—C11	122.22 (19)
C9—C8—H8	120.0		
			
B—N2—C1—C2	−179.4 (2)	C6—C5—C10—C1	178.8 (2)
B—N2—C1—C10	0.1 (3)	C2—C1—C10—C9	179.5 (2)
N2—C1—C2—C3	−179.5 (2)	N2—C1—C10—C9	−0.1 (3)
C10—C1—C2—C3	1.0 (3)	C2—C1—C10—C5	−0.4 (3)
C1—C2—C3—C4	−1.3 (4)	N2—C1—C10—C5	−179.92 (19)
C2—C3—C4—C5	1.1 (4)	C16—C11—C12—C13	−0.9 (4)
C3—C4—C5—C6	−179.1 (2)	B—C11—C12—C13	177.9 (2)
C3—C4—C5—C10	−0.5 (3)	C11—C12—C13—C14	−0.3 (4)
C4—C5—C6—C7	179.6 (2)	C12—C13—C14—C15	1.5 (4)
C10—C5—C6—C7	0.9 (3)	C12—C13—C14—Cl	−178.80 (19)
C5—C6—C7—C8	−0.2 (4)	C13—C14—C15—C16	−1.4 (4)
C6—C7—C8—C9	−0.4 (4)	Cl—C14—C15—C16	178.9 (2)
C7—C8—C9—N1	−179.2 (2)	C14—C15—C16—C11	0.2 (4)
C7—C8—C9—C10	0.2 (3)	C12—C11—C16—C15	0.9 (4)
B—N1—C9—C8	179.2 (2)	B—C11—C16—C15	−177.8 (2)
B—N1—C9—C10	−0.2 (3)	C1—N2—B—N1	−0.2 (3)
C8—C9—C10—C5	0.5 (3)	C1—N2—B—C11	177.6 (2)
N1—C9—C10—C5	179.96 (18)	C9—N1—B—N2	0.2 (3)
C8—C9—C10—C1	−179.3 (2)	C9—N1—B—C11	−177.60 (19)
N1—C9—C10—C1	0.1 (3)	C12—C11—B—N2	178.0 (2)
C4—C5—C10—C9	−179.77 (19)	C16—C11—B—N2	−3.3 (3)
C6—C5—C10—C9	−1.1 (3)	C12—C11—B—N1	−4.3 (3)
C4—C5—C10—C1	0.1 (3)	C16—C11—B—N1	174.4 (2)

### Hydrogen-bond geometry (Å, °)

**Table:**

*D*—H···*A*	*D*—H	H···*A*	*D*···*A*	*D*—H···*A*
N2—H2···Cl^i^	0.78 (2)	2.93 (2)	3.666 (2)	158 (2)

Symmetry codes: (i) −*x*−1, *y*+1/2, −*z*+1.
